# Artificial Intelligence in Obstetrics and Gynaecology: Advancing Precision and Personalised Care

**DOI:** 10.7759/cureus.86929

**Published:** 2025-06-28

**Authors:** Nida Aftab

**Affiliations:** 1 Obstetrics and Gynaecology, Aneurin Bevan University Health Board, NHS Wales, Newport, GBR

**Keywords:** artificial intelligence (ai), clinical decision-making, clinical research productivity, healthcare innovation, obstetrics and gynaecology (obs/gyn), predictive analytics & ai, robotic assistance surgery, simulation of digital health data, ultrasound imaging, workflow efficiency

## Abstract

Artificial intelligence (AI) is rapidly transforming the landscape of obstetrics and gynaecology, offering unprecedented capabilities in diagnostics, monitoring, and personalised treatment. This review highlights the integration of AI in various domains, including obstetric imaging, fetal monitoring, gynaecologic oncology, fertility treatment, and robotic surgery. AI-powered tools are shown to enhance precision by improving diagnostic accuracy, reducing human error, and supporting clinical decision-making. Importantly, the article explores the global implications of AI adoption, including applications in low-resource settings, and emphasises the need for ethical considerations, data inclusiveness, and clinician trust. Overall, this comprehensive review demonstrates how AI is enabling more individualised and effective care in women's health.

## Introduction and background

Artificial intelligence (AI), particularly machine learning (ML) and deep learning, is transforming healthcare by enabling computers to interpret complex medical data [1]. In obstetrics and gynaecology, AI has the potential to enhance screening, diagnosis, and management by extracting patterns from large datasets. For example, AI algorithms can analyse medical images (ultrasound and MRI) to detect subtle anatomical structures or anomalies that may be missed by human observers [2]. Radiology, a key diagnostic tool in women’s health, is one area where AI (especially convolutional neural networks (CNNs)) has already shown great promise in image classification and segmentation. Beyond radiology, AI applications in obstetrics and gynaecology span a broad range of areas -- from prenatal imaging and fetal heart rate monitoring to gynaecologic cancer screening, reproductive medicine (fertility and in-vitro fertilisation (IVF)), robotic surgery, and clinical decision support systems. In general medicine, only a few AI-based devices had been FDA-approved by 2020 [1]; however, research interest is surging, and the number of AI publications in obstetrics and gynaecology journals has grown markedly [1]. Precision and personalised care are especially important in obstetrics and gynaecology, since each pregnancy or patient's history is unique. For example, accurate estimation of gestational age or individual risk of preterm birth allows tailored prenatal management. In gynaecology, identifying individual tumour risk or selecting optimal fertility treatments requires personalising decisions. AI supports these goals by integrating diverse data (imaging, clinical, genomic, and wearable sensors) and delivering patient-specific predictions. By improving diagnostic accuracy and consistency, AI can help ensure timely interventions and reduce adverse outcomes for both mother and child [1].

This review aims to systematically evaluate the current applications of AI across obstetrics and gynaecology and assess how these technologies contribute to precision diagnostics and personalised care in women's health. Specifically, the objectives of this review are: (i) to provide a comprehensive overview of AI use cases in major subfields of obstetrics and gynaecology; (ii) to present representative examples and performance metrics of AI-driven tools in these domains; and (iii) to discuss the implications of these developments and how our review extends insights from recent literature.

## Review

Methods

A literature search was conducted to gather relevant studies on artificial intelligence (AI) in obstetrics and gynaecology. We queried databases (PubMed, Scopus, and Web of Science & internet web) up to May 2025 using keywords such as “artificial intelligence,” “machine learning,” “deep learning,” “obstetrics,” “gynaecology,” “pregnancy,” and “women’s health.” The search was restricted to English-language publications. Articles were included if they reported or reviewed applications of AI (including ML and deep learning models) in obstetric or gynaecologic practice. This encompassed clinical studies, systematic reviews, and proof-of-concept trials across imaging, fetal monitoring, risk prediction, treatment, and surgery. We excluded studies focusing on unrelated fields or lacking substantive data on AI performance. Titles and abstracts were screened for relevance, and the full texts of eligible papers were reviewed to extract details on the AI methodology, study population, and key outcomes. Data from the selected articles were synthesised narratively, focusing on how AI techniques were applied and their efficacy compared to standard care. Consistent terminology was used throughout (e.g., adopting British English spelling of gynaecology and related terms). All abbreviations have been expanded at first mention and are used consistently thereafter in the manuscript.

AI in Obstetric Imaging and Diagnostics

AI is being applied to standard prenatal imaging to improve objectivity and efficiency. Advanced deep learning models can automatically measure fetal biometry from ultrasound images, often matching or exceeding human accuracy [3]. One study demonstrated an AI model that performed entire fetal scans and computed biometry (biparietal diameter, head/abdominal circumference, femur length, etc.) without manual input, potentially reducing scan time by up to one-third [3]. The model produced measurements as reliable as those obtained by expert sonographers and was robust across different machines and operators. In practice, such automation could standardise measurements and free sonographers to focus on interpretation or patient communication. Beyond basic biometry, AI aids prenatal anomaly detection. Large clinical datasets are used to train convolutional neural networks (CNNs) to recognise patterns of congenital malformations. For example, a convolutional U-Net combined with a Visual Geometry Group (VGG) network was used to detect central nervous system (CNS) defects (e.g., neural tube defects, ventriculomegaly) on fetal brain ultrasound. This system reduced false negatives for brain anomalies by 97.5%, dramatically improving sensitivity [4]. The same model achieved ~96-97% correct classification of normal versus abnormal fetal brain anatomy [4]. Similarly, real-time deep learning systems, e.g., the prenatal ultrasound diagnosis artificial intelligence conduct system (PAICS) demonstrated high diagnostic accuracy for fetal central nervous system (CNS) abnormalities in both image datasets and real-time scans. Comparable to expert performance but more time-efficient, this CNN-based tool shows strong potential for effective clinical screening of fetal CNS malformations [5]. These AI tools match expert-level performance and can shorten scanning time, suggesting they may serve as decision support in busy clinics. Artificial intelligence also assists in more complex imaging like fetal MRI. In placenta evaluation, for instance, ML-based texture analysis of MRI can help predict conditions like placenta accreta (though this research is still emerging). In general, AI’s ability to integrate ultrasound and MRI features holds promise for better risk stratification of obstetric conditions (e.g., placenta previa, twin-to-twin transfusion syndrome). A recent review noted that AI excels at analysing multimodal imaging data, automatically identifying structures such as the placenta or fetal organs and even predicting outcomes (e.g., preterm birth) from imaging combined with clinical records [6]. Thus, AI enhances prenatal diagnostics by making image interpretation faster and more standardised.

AI in Fetal Monitoring and Risk Prediction

Continuous monitoring of fetal well-being and maternal risk is a fertile area for AI. Electronic fetal monitoring (cardiotocography (CTG)) generates large streams of fetal heart rate and uterine contraction data. However, CTG interpretation by humans suffers high inter-observer variability. AI-driven analysis of CTG can reduce subjectivity: for example, a team trained a deep learning model on nearly 19,400 CTG recordings matched with cord blood pH and Apgar outcomes [7]. The AI model was designed to predict fetal hypoxia, and preliminary results suggest it could provide consistent CTG interpretation to alert clinicians before distress. In a separate study, deep CNN models for CTG achieved performance similar to expert clinicians (area under the curve (AUC) ~0.68-0.70) and highlighted the feasibility of algorithmic fetal compromise prediction [8]. Such models aim to flag high-risk CTG patterns, potentially reducing missed cases of fetal distress. AI is also used for predicting obstetric complications from routine clinical data. For example, multiple machine learning models (random forests, gradient boosting, elastic net, etc.) have been developed to predict pre-eclampsia from prenatal data. A recent systematic review found ML models yielding high accuracy for pre-eclampsia risk, with area under the curve (AUC) values ranging from 0.86 to 0.97 across studies [9]. These models incorporate maternal history, laboratory results, and even early ultrasound markers to estimate risk. Similarly, AI algorithms have been applied to predict outcomes like preterm birth, gestational diabetes, and postpartum haemorrhage, often outperforming traditional statistical scores. Mental health after childbirth is another emerging AI application. A U.S. study trained a machine learning model on electronic health records (EHRs) of 29,000 mothers to predict postpartum depression. Individuals flagged as high-risk by the model were nearly three times as likely to develop the condition compared to the average [10]. This kind of risk stratification could allow targeted psychological support. In general, AI-enabled risk calculators in obstetrics -- from fetal distress to psychiatric outcomes -- promise more personalised surveillance.

AI in Gynaecologic Imaging and Cancer Screening

Gynaecologic imaging is likewise benefiting from AI. For example, ovarian masses are routinely evaluated by transvaginal ultrasound. A recent large multicentre study with 17,119 images (3652 patients across eight countries) trained transformer-based neural networks on ultrasound images to distinguish benign from malignant ovarian lesions. The AI models generalised well across centres and systems, significantly outperforming both expert and novice clinicians on sensitivity, specificity, and overall accuracy [11]. The same study reported that in a simulation of clinical triage, the AI reduced referrals to specialists by 63% while maintaining diagnostic quality [11]. This international validation shows that AI could alleviate shortages of expert sonographers and improve early cancer detection in gynaecology.

Similarly, AI is revolutionising cytology and pathology in women’s health. One Nature Communications study developed an AI system (AICCS) for cervical cytology screening. Trained on >16,000 cases, the AI achieved very high performance in classifying Pap smear images. In a prospective trial, the AI system alone reached an AUC of ~0.947 for detecting high-grade lesions [12]. When used to assist cytopathologists, the AI significantly improved their diagnostic sensitivity by ~13.3% [12]. Such systems suggest that AI can act as a digital second reader in cancer screening, increasing accuracy and consistency. Beyond cancer, AI tools assist routine gynaecological imaging. For instance, AI algorithms are being trained to segment uterine fibroids or the endometrial lining on ultrasound and MRI, quantify their volume, and even predict response to medical therapies. In gynaecologic oncology, AI “radiomics” approaches analyse patterns of tissue texture on MRI to estimate tumour aggressiveness. While these fields are evolving, the trend is clear: AI-driven imaging analysis can extract quantitative biomarkers from scans that personalise diagnosis and treatment planning.

Robotic and Assisted Surgery With AI

Robotic surgery is widely used in gynaecology (e.g., robotic hysterectomy, myomectomy) for its precision and minimally invasive approach. Modern robotic platforms (such as the da Vinci system) already integrate advanced software control; the next step is embedding machine learning. AI can enhance robotic surgery by providing real-time analytics and guidance. For example, AI algorithms can process endoscopic video feeds to identify anatomy or pathology, alerting the surgeon to critical structures (vessels, nerves, and tumours) and enhancing safety. In training and evaluation, ML models have been developed to assess surgical skills from instrument motion data, offering objective feedback to surgeons. A recent overview noted that "the integration of AI and ML into robotic systems holds great potential," since AI can supply real-time data analysis, predictive analytics, and decision support to improve precision and outcomes of robotic-assisted procedures [13]. In practice, research is ongoing. Some groups are exploring “semi-autonomous” robotic features (e.g., AI-controlled suturing or camera guidance) under surgeon supervision. Even without full autonomy, AI modules can optimise ergonomics (automatically adjusting the camera angle) or coach novice surgeons via augmented reality. As the technology matures, we expect to see AI-driven enhancements in laparoscopic gynaecology, making complex surgeries safer and more reproducible.

Artificial Intelligence in Fertility and Reproductive Health

Reproductive medicine is another area of AI innovation. In in-vitro fertilisation (IVF), embryologists currently assess embryo quality by eye -- a subjective process. AI offers more objective embryo selection. A recent study applied CNNs to time-lapse images of embryos and found that an AI model predicted pregnancy success with ~75% accuracy, outperforming experienced embryologists [14]. Using multiple image frames (early, mid, and late development) further improved prediction. By analysing subtle morphological and developmental patterns invisible to the human eye, AI-assisted embryo selection could raise implantation rates and reduce the number of cycles needed per pregnancy. AI is also used for cycle tracking and ovulation prediction. Smartphone apps and wearable sensors feed ML algorithms (e.g., on basal body temperature, heart rate, and hormone levels) to predict fertile windows more accurately than calendar methods. Personalised dosing of hormones for ovarian stimulation in IVF is another target: ML models can suggest optimal medication regimens based on patient-specific data (age, anti-Müllerian hormone levels, past responses), moving towards a precision-medicine approach.

Clinical Decision Support Systems (CDSS)

Across obstetrics and gynaecology, AI is increasingly embedded in clinical decision support systems. These CDSS tools ingest electronic health record data, diagnostics, and guidelines to provide evidence-based recommendations or risk alerts at the point of care. A systematic review identified 30 studies of AI-augmented CDSS in pregnancy, covering the prenatal period through postpartum [15]. The CDSS functions ranged from diagnostic support (e.g., automated anomaly detection) to clinical prediction (e.g., estimating preterm birth or pre-eclampsia risk) and therapeutic guidance. Examples include AI models integrated into ultrasound machines to flag abnormal findings in real-time, or EHR-based alerts that identify women at high risk for conditions like gestational diabetes or fetal growth restriction. Lin et al. noted that advances have been made in “early diagnosis of prenatal abnormalities” and “prenatal ultrasound support” through AI-CDSS [15]. Some systems incorporate patient-specific factors to tailor recommendations (for instance, adjusting the schedule of fetal monitoring for women with multiple risk factors). Importantly, many AI-CDSS tools under development focus on improving surveillance efficiency (e.g., predicting who needs more frequent visits) and knowledge integration (e.g., linking genomic or social determinant data).

Overall, AI-based CDSS promises to make obstetric and gynaecologic care more proactive and personalised. For example, a CDSS might combine maternal demographics, ultrasound results, and lab values to calculate a personalised risk score for pre-eclampsia, prompting earlier aspirin prophylaxis if indicated. Such tools are still emerging, and researchers emphasise the need for rigorous validation. Nevertheless, preliminary evidence suggests that AI-CDSS can improve diagnostic accuracy and patient outcomes [15] when properly implemented.

AI in Global and Low-Resource Settings

AI also has global health implications. In low-income countries where skilled sonographers are scarce, AI-powered portable ultrasound could democratise prenatal care. For instance, a collaborative study in Zambia and the USA developed a mobile AI model that estimated gestational age and fetal lie from “blind sweep” ultrasound scans acquired by novices. The AI’s gestational age estimates were statistically non-inferior to standard biometry (mean error ~4.5 days) even when scans were done by minimally trained users [16]. The same model detected malpresentation (breech vs head-first) with very high accuracy (AUC 0.977). Such tools could allow frontline midwives to provide higher-quality obstetric assessments without requiring expert technicians, thus expanding access to precision prenatal dating and identifying complications earlier.

Health Care Professionals’ Concerns About Medical AI 

Overcoming resistance to medical AI seems to hinge on thorough education, the establishment of appropriate regulations, and clearly defined roles. Tackling these areas may encourage greater acceptance and more effective integration of AI [17].

## Conclusions

Artificial intelligence is rapidly advancing precision and personalised care in obstetrics and gynaecology. Across imaging, monitoring, surgery, fertility, and decision support, AI-based tools have demonstrated higher accuracy and efficiency than traditional methods. For example, deep learning ultrasound systems can measure fetal growth and detect congenital anomalies with expert-level performance. While AI-driven cytology screening yields near-human accuracy in cervical cancer detection. Robotic surgeries enhanced by AI promise even greater precision and standardisation in complex procedures. In fertility medicine, ML models can objectively select the most viable embryos to improve IVF success rates. Overall, AI can integrate diverse patient data to tailor prevention and treatment -- fulfilling the goals of precision medicine in women’s health. Despite these benefits, key challenges remain. AI models are only as good as their data: biases in training datasets, such as the underrepresentation of certain populations, can lead to unequal performance. Studies have noted gaps in AI-CDSS validation and potential biases from social determinants of health. Clinician acceptance is another hurdle; obstetricians and gynaecologists must trust and understand AI recommendations. The regulatory environment is still catching up, as evidenced by the relatively small number of approved AI tools in this field. Data privacy and integration with electronic health records also pose logistical barriers. Finally, most AI systems need extensive prospective clinical trials before widespread adoption.

Future directions involve addressing these challenges. Multicentre international studies, such as the multi-country ovarian ultrasound validation, are important to ensure generalisability. There is also a push to develop interpretable AI that provides a rationale (not just black-box output) to clinicians. Integration of genomics and wearable data with obstetric AI models may further enhance personalisation. Importantly, obstetric and gynaecologic practitioners should be involved in AI development to align tools with clinical workflows and patient needs. In summary, AI stands to greatly improve maternal and neonatal outcomes by enabling truly personalised care -- provided that its development remains patient-centred, transparent, and ethically grounded.
